# Student participation in governance of medical and veterinary education: experiences and perspectives of student representatives and program directors

**DOI:** 10.1007/s10459-019-09890-9

**Published:** 2019-05-01

**Authors:** Stephanie N. E. Meeuwissen, Annemarie Spruijt, Jeroen W. van Veen, Anton F. P. M. de Goeij

**Affiliations:** 1 Department of Educational Development and Research, Faculty of Health, Medicine and Life Sciences, Graduate School of Health Professions Education, Maastricht University, P.O. Box 616, 6200 MD Maastricht, The Netherlands; 2 Faculty of Veterinary Medicine, University of Utrecht, Utrecht, The Netherlands; 3 Department of Respiratory Medicine, Zuyderland MC, Heerlen, The Netherlands; 4 Institute for Education, Faculty of Health, Medicine and Life Sciences, Maastricht University, Maastricht, The Netherlands

**Keywords:** Education management, Medical education, Student engagement, Student participation, Student representatives, Student-staff collaboration, Student-staff partnership, Student voice, University governance, Veterinary education

## Abstract

Student participation in governance of education is of growing interest. However, it remains unclear what factors render this participation in institutional governance a success or a failure. Another question is: what are the perceived benefits for schools and students? We empirically explored experiences and perspectives of student representatives and program directors of all (8) medical and (1) veterinary schools in the Netherlands on factors that influence student participation in institutional governance and its values and challenges for schools and student representatives. A constructivist grounded theory study was performed. A theoretical sample of student representatives was invited to fill out an explorative, qualitative questionnaire. Next, focus groups with student representatives and interviews with all program directors were conducted. Data was analyzed using open, axial and selective coding by all authors. Experiences and perspectives of students and program directors were remarkably similar in both perceived influences and values. Four main categories of influences could be distinguished in student participation: (1) individual student characteristics, (2) individual staff characteristics, (3) the organization of student representatives and (4) the school’s organization, including its culture and policy regarding student participation. A cohesive, well-organized and independent student organization has crucial impact on student participation in educational governance processes. For representatives, major benefits of participation are personal and career development. Challenges are low effectiveness and efficiency of their actions. A clear school policy on student participation and better introduction, feedback and coaching of representatives should be provided to improve student participation in governance processes.

## Introduction

Since the introduction of student-centered approaches, students have been engaged in their education. However, for a long time, this has been limited to filling out questionnaires on program evaluation. Occasionally, interested students could participate in committees by collaborating with staff on course content and curriculum development. A growing body of evidence clearly demonstrates that medical students contribute significantly to quality and content of their education program (Anderson 2006; Dhaese et al. 2015; Furmedge et al. 2014). Student engagement encompasses a wide range of collaborative activities with staff in universities, which enhance student learning and development, and contributes to improve the quality of academic environment and culture in the institutes (Peters et al. 2018; Trowler 2010). These collaborative activities may include either engagement of the whole student population or student representatives (Bovill et al. 2016).

Various forms of student engagement have developed, such as student-staff collaboration in learning, teaching and research (Bovill et al. 2016; Dickerson et al. 2016; Trowler 2010), co-creation in design of educational programs using student representation systems (Bovill et al. 2016; Yengo-Kahn and Baker 2017) and participation of student representatives in university governance and decision-making (Visser et al. 1998; Carey 2013, 2018; Lizzio and Wilson 2009; Planas et al. 2013; Trowler 2010). Several conceptual frameworks for student engagement in higher education have been presented (Carey 2018; Kahu 2013; Lizzio and Wilson 2009). Increasing levels of student engagement are recognized and vary from consultation, involvement, and participation to partnership (Cook-Sather 2014; Healey et al. 2014). In consultation, students can express their perspectives, whereas in involvement, they can take a more active role. Student participation can be described as an active role in a defined, collaborative process with staff, while student-staff partnership is defined as joint ownership and decision-making over processes and outcomes (Healey et al. 2014).

In higher education, there is a growing interest in student engagement, described as the ‘student voice’, student participation, student-staff partnership, student representation and student power (Bovill et al. 2016; Carey 2018; Klemenčič 2012, 2014). This interest is shown e.g. by activities of the Higher Education Academy in the UK (Healey et al. 2014). Also, a call was launched by the International Association for Medical Education in Europe (AMEE) for recognition of excellence in medical, dental and veterinary schools for student engagement in management, the education program and the academic community (ASPIRE initiative—International Association for Medical Education in Europe 2012). This was recently overviewed by Peters et al. (2018). Moreover, the extent of student engagement in higher education is increasingly a quality criterium in accreditation of education programs in Europe and Australia (Carey 2013; Peters et al. 2018). In the World Federation of Medical Education (WFME) global standards for quality improvement, student engagement in medical curricula is now even a basic tenet (WFME Global Standards For Quality Improvement 2015).

Evidence has been reported for beneficial effects of enhanced student-staff collaboration in education, such as improved meta-cognitive understanding of learning and teaching processes, a better quality culture and organizational learning, and development of a range of graduate attributes (Bendermacher et al. 2017; Bovill et al. 2016; Carey 2013; Cook-Sather 2014). A driving force for students to participate in governance is presumably their growing interest in developing competencies, e.g. communication and leadership skills, contributing to personal and professional development and expanding their extracurricular experiences. Furthermore, an increased awareness among program directors, course coordinators and students can be observed that successful student-staff collaboration may have interesting and unique benefits for the education program as well as for the stakeholders involved.

However, student participation and collaboration between students and staff does not always go smoothly and effectively in practice (Bovill et al. 2016; Lizzio and Wilson 2009; Stalmeijer et al. 2016). In the medical education realm, student representatives experienced difficulties in providing feedback to teachers (Griffin and Cook 2009; Stalmeijer et al. 2016) and felt a lack of appreciation (Lizzio and Wilson 2009).

In the higher education system of the Netherlands, students are extensively engaged in institutional governance. Our interest in experiences and perspectives of stakeholders in governance of education was particularly raised when conducting a workshop organized by the national student representation platform in which student representatives from all Dutch medical and veterinary schools participated. The attending students were engaged as representatives in quality assurance or governance of education in their respective schools. The students reported both benefits and challenges and, most importantly, they expressed their concern over the extent to which their input was actually heard and acted upon in institutions’ processes, a sentiment which finds resonance in previous study findings (Little and Williams 2010). This triggered us to study these issues in-depth, by involving the stakeholders in governance.

Reports on processes and benefits of student participation in governance are scarce, and also the conditions and settings of these studies differ significantly from the context of medical and veterinary education. In order to get more insight in the process and the outcome of student participation in governance of education we empirically explored the experiences and perspectives of student representatives and program directors.

The present study addresses the following research questions.

(1) what factors render student participation in medical and veterinary education governance successful or not, and (2) what is the perceived value of student participation for the education program, the school and its student representatives?

## Methods

### Setting and context

This study was conducted in all medical (N = 8) and veterinary (N = 1) schools in the Netherlands from May to August 2016. The schools have respectively CanMEDS (Frank and Danoff 2007) or VetPro (Bok et al. 2011) competency-based curricula with three years bachelor’s and three years master’s programs (Ten Cate 2007). In the Netherlands, student representatives are able to participate in governance of higher education, which includes quality assurance, management and decision-making. Student engagement is legally enshrined in the Higher Education and Research Act which specifies that students should be represented at three levels: the university council, the faculty council or student council and the education committee of their curriculum (Act on Higher Education and Scientific Research (in Dutch: Wet op het hoger onderwijs en wetenschappelijk onderzoek, WHW 1992). Each medical and veterinary school has appointed a student assessor who represents the students at the highest level and who is in direct contact with the program director and sometimes the dean or even the executive board of the university medical centre. For more information on the positioning and roles of Dutch student representatives see “Appendix 1”.

The program director is primarily responsible for the governance of the six-year curriculum, and leads the school’s management team, which usually consists of at least the coordinators of the bachelor’s and the master’s programs and a student advisor.

### Participants

Participants were medical and veterinary student representatives, and all medical and veterinary program directors. Students who were currently functioning or had recently been active as representative during more than three months were included. In this way, students were sampled with a range of student representatives’ roles and experiences.

All eight medical program directors and both the present and the former program director of the veterinary school in the Netherlands accepted our invitation and participated in the study.

### Methodology

This study followed a constructivist grounded theory methodology to explore experiences and perspectives in student-staff collaboration in medical and veterinary education (Charmaz 2008; Watling and Lingard 2012). We thus acknowledged that existing experiential and theoretical background (ASPIRE initiative—International Association for Medical Education in Europe 2012; Healey et al. 2014) in student participation and collaboration with staff was present among all members of the research team. A qualitative multi-centre, multimethod approach, triangulating multiple data sources and data gathering techniques, was used (Creswell 2015).

### Procedure

#### Questionnaires and focus groups with student representatives

According to Watling and Lingard (2012) we theoretically sampled the student assessor of each medical and veterinary school. Subsequently, we asked the student assessors to invite a heterogeneous group of student representatives in their own respective schools to fil out an explorative questionnaire. This questionnaire probed the representatives’ experiences and was based on conceptual ideas drawn from experiences and current literature on student participation and student-staff collaboration (ASPIRE initiative—International Association for Medical Education in Europe 2012; Healey et al. 2014). Responses from the questionnaires were used to refine the focus group interview guide and used as sources to stimulate in-depth focus group discussions. Questionnaire topics included school’s organization and student-staff collaboration, specifically efficiency, effectiveness, boundaries, difficulties and successes. See “Appendix 2”. Although the ultimate goal of the questionnaire was to explore topics for the focus group discussions, the frequency of the issues raised by students was determined to indicate their perceived importance. All students who had completed the questionnaire were invited to attend a focus group. This approach was chosen to allow participants to share their experiences, complement ideas and make collective sense (Barbour 2008).

Focus groups were guided by a semi-structured discussion protocol that addressed the same topics as those included in the questionnaire. Before starting the focus group series, a dry run was performed that led to improvements of the discussion protocol. Focus group sessions lasted 60–90 min. Each focus group consisted of six student representatives. Focus groups were moderated by both a student and a staff member of the research team, acting in rotation. Focus groups were held until after analyzing the fifth focus group, the categories were adequately understood, we did not find new concepts or relationships and the research team reached consensus that theoretical saturation was achieved (Watling and Lingard 2012).

#### Interviews with program directors

Program directors were invited for a personal interview by email. The topics of the interview guide were constructed in a similar way as those of the student questionnaire and discussion protocol. Since we used an iterative approach, the guide took further shape in the course of the interviews, although the key questions remained unaltered. The directors received the questions prior to the interview in order to prepare, if necessary, with the help of their staff members. Interviews lasted approximately 60 min and were conducted by two alternating members of the research team, all of whom had considerable experience with interviewing. The research team concluded that after nine interviews theoretical saturation was achieved by the collection of rich data, combining both positive and negative cases. However, we decided to interview the remaining program director and explicitly sought for contradicting perspectives and experiences to test the developed coding framework. This interview supported the coding framework. All focus groups and interviews were audiotaped and the ensuing audio fragments transcribed verbatim by a professional transcription service.

### Data analysis

#### Questionnaires and focus groups

The frequency of issues raised by students in the questionnaire was determined, categorized and related to the response rate. AdG and JvV coded the focus group transcripts independently and discussed the preliminary coding framework after every two focus groups. They followed the stages of open and axial coding, discussed the codes and constructed categories and subcategories in an analytic process of constant comparison (Corbin and Strauss 1990; Watling and Lingard 2012). After the analysis of the fifth focus group, AS read and coded 20% of all focus group transcripts so as to eventually confirm the coding framework.

#### Interviews

As with the focus groups, the stages of open and axial coding were followed (Corbin and Strauss 1990). SM and AS coded the interview transcripts independently, discussing their findings after every two interviews until they reached consensus on codes. After nine interviews, they discussed codes and categories until they reached consensus and refined the coding framework. Subsequently, researcher triangulation was applied by submitting the coding framework to AdG who confirmed it after reading all interviews and discussing and refining code names.

#### Analysis of all data

Questionnaire data were used in data triangulation with focus groups and interviews. This is exemplified in Table 1. The data obtained from questionnaires and focus groups were compared with those resulting from interviews to be able to make tentative suggestions for the influencing factors and values of student participation. All researchers were involved in this stage of selective coding (Corbin and Strauss 1990). Afterwards, AdG reread all transcripts to make sure that no relevant information had been missed.

**Table 1:** Example of data triangulation with respect to attitudes of individual students, e.g. being pro-active and prepared

Source of data	Individual student attitudes: being pro-active and prepared
Questionnaire students	*“During meetings they* [staff] *sometimes express their appreciation for students’ opinions and thanks for our* [students] *valuable preparation or input.” (Q*-*11)* *“The thing that has led to our success is mainly, I think, being assertive at the right time and especially our commitment to establishing a collaboration. We always try to create an atmosphere where we can work on a solution together, instead of creating a toing and froing scenario.” (Q*-*7)*
Focus group students	*“Yes, I do believe that, preparation is an important point as well. […] Because it is a job that requires action on your part and it would be wrong to think that the program just drops it into your lap each time, like: ‘deal with this problem’; you too must actively solve problems yourself.” (FG*-*1)*
Interview program directors	*“The better you* [a student] *back up your opinion with arguments and prepare your documents, the better the contact with your fellow students will be; this empowers you as a student to perform well.” (Int*-*4)*

Quotes are taken from Questionnaires (Q), Focus Groups (FG), and Interviews (Int)

For purposes of enhanced coherence and legibility, quotes, questionnaires and interview scripts have been subject to light editing before journal submission. The authors, however, based their analysis on the original, untidied-up transcriptions, questionnaires and scripts. If necessary, the original quotes can be requested from the first author

### Ethical considerations

Approval for this study was obtained from the Netherlands Association for Medical Education Ethical Review Board (NVMO ERB 699). All participants were informed that participation was voluntary and all gave informed consent. To preserve anonymity, we coded participants’ names and de-identified all the quotes used in this article.

## Results

### Characteristics of participants

The explorative, qualitative questionnaire was completed by 43 student representatives recruited from all medical schools and the only veterinary school in the Netherlands. The five focus groups were attended by a total of 30 students from six different schools, representing all six years of the respective education programs. Interviews were conducted with 10 program directors. Please see Table 2 for demographics.

**Table 2:** Demographics student questionnaires, student focus groups and interviews with program directors

Participants	Questionnaire students (n = 43)	Focus groups students (n = 30)	Interviews program directors (n = 10)
**Gender**			
Men	13	8	8
Women	30	22	2
**Program**			
Bachelor	22	18	n/a
Master	21	12	

### Factors that affect student participation and collaboration with staff

**Fig. 1:**
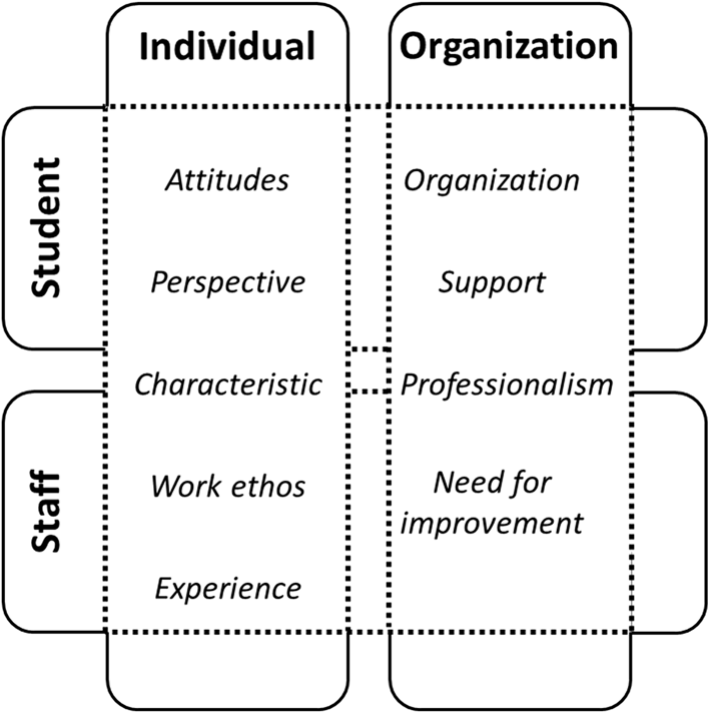
Perceived factors affecting student participation

By addressing our first research question, four main categories, revealing both positive and negative factors, were constructed from the analysis of the stakeholders’ experiences and perspectives: (1) individual student characteristics, (2) individual staff characteristics, (3) the organization of the student representatives and (4) the school’s organization, including its culture and policy regarding student participation. For these categories, a number of subcategories have been identified. See Fig. 1.

#### Individual student characteristics

Program directors and students shared the view that specific attributes of students influenced the success of student-staff collaborations in governance. Students with a proactive and critical attitude, particularly those who had a long-term perspective were seen as the ones who fared best in student-staff collaborations. In addition, being adequately prepared for meetings and informed by keeping close contact with fellow students to know what is going on in practice, were qualities that participants of both groups of stakeholders considered fundamental.

Another student characteristic mentioned repeatedly in focus groups was a sense of responsibility towards their tasks in medical education, besides showing self-confidence and diplomacy. Similarly, a broad perspective and an open mind figured prominently in both focus groups and interviews. Program directors pointed out that students who were most successful typically had some experience with the educational organization, were flexible, quick learners, worked with respect for others and, rather than pursuing their own interest, were in it for the common good. In the questionnaire, a majority of the responding representatives (81%) mentioned improvement of education as their motivation, which is in line with the latter observation by the program directors.

Worthy of mention are the assertions that student characteristics conducive to effective collaboration also depend on the type of role a student fulfils.

These influencing factors and corresponding quotes are summarized in Table 3.

**Table 3:** Perceived factors influencing student participation: individual student and staff

Individual student		
Attitudes	Proactive Critical Flexible Respectful	*“Yes, I do believe that preparation is an important point as well, […] it is a job that requires action on your part. […] You too must actively solve problems yourself.” (FG*-*1)*
Perspective	Open mind Long-term and Broad perspective Be in it for the common good	*“That one is able to view things from a 360*-*degree perspective, to consider the interest of students, the interest of the organization and the interest of content* –*that is what I consider really important, that they are able to take stock of that whole picture. That they are able to set priorities, that they remain calm and defend their opinion, not their own opinion, but their collective opinion. So they are not there to pursue their own interests, I have experienced that a couple of times too, that this was the case. I disapprove of that.” (Int*-*5)*
Characteristic	Self-confidence Diplomacy Quick learner Profile fits role	*“You have to think, balance different opinions, reach a conclusion or decision. A bit of diplomacy […] and you have to have […] ideals or a clear goal, but you must also be able to realize that you cannot always reach every single goal and that sometimes you must give up one battle for the sake of winning another. You must be able to cope with adversity.” (FG*-*4)* *“Then you are talking about a completely different role* [assessor] *than when you talk about a member of the student representatives’ platform, and within a student representatives’ platform you need a variety of people. So I feel that if you have a few people in it who are very silent and great thinkers. That can be really helpful. Then those may not be the first ones you send to the Executive Board, but you do need them too.” (FG*-*4)*
Work ethos	Sense of responsibility Being prepared Well-informed by peers	*“The better you* [a student] *back up your opinion with arguments and prepare your documents, the better the contact with your fellow students will be; this empowers you as a student to perform well.” (Int*-*4)*
Experience	With educational organization	*“Many of our representatives are functioning during several years, so we have people in our* [student] *board with experiences and contacts. That makes* [successful representation] *easier.” (Q*-*35)*

Individual staff		
Attitudes	Willing to work with students Willing to compromise Taking students seriously	*“Well yes, I don’t take that* [suggestion raised by students] *to all those staff members, you know; the ones that I think, well, I don’t need to tell you that this is what the students want, I will just say that this is what I want.” (Int*-*2)* *“Yes, because for instance I personally consider the issue of […] an important topic, but it is not high on the agenda. And since it is not listed high on their agenda, you can […] try to persuade as loud as you want to, it is not going to happen.” (FG*-*4)*
Perspectives	Be in it for continuously improving education	*“To make clear to coordinators and teachers the importance of involving students: that it makes no sense to develop something that has not been seen by any student. […] Also to make clear to the teacher, ‘you are spilling a lot of your precious time and you are making it inefficient. Use the available expertise; students will not slate it, but will primarily look how it may be improved’.” (Int*-*1)*
Characteristic	Knowing the student organization	*“It is counterproductive, so that is what I explained to him: ‘All documents must be accessible to them, they really should have a sense of belonging, otherwise, sooner or later, there will be a problem.” (Int*-*10)*
Work ethos	Dedicated to education	*“There are quite some people at high positions around here, who want the very, very, very best for students. I do find that a strong vision. These people find it very important that students participate, they like to see them everywhere, at all fields from policy to governance to practice.“(FG*-*3)*
Experiences	As a student representative	*“I used to be a student representative myself. I remember that we organized a conference in our first year, and in year five we invited the Secretary of State* - *and he came! So yes, of course, student participation is important.” (Int*-*1)*

Quotes are taken from Questionnaires (Q), Focus Groups (FG), and Interviews (Int)

For purposes of enhanced coherence and legibility, quotes, questionnaires and interview scripts have been subject to light editing before journal submission. The authors, however, based their analysis on the original, untidied-up transcriptions, questionnaires and scripts. If necessary, the original quotes can be requested from the first author

#### Individual staff characteristics

According to both student representatives and program directors successful collaboration was most likely when dealing with staff who were dedicated to continuously improving the quality of education. In particular, staff’s willingness to work with students and taking students’ opinions and contributions seriously. were flagged as important markers of success. This experience was shared by both students and program directors. If this attitude was lacking, participation and collaboration were hampered or even blocked. As a remedy, one of the program directors would present the students’ opinion as her own to colleagues whom she believed were not responsive to students. Occasionally, program directors redressed a negative attitude of staff members towards student representatives. This is illustrated in the case of a course coordinator who was not willing to provide information to student representatives on a curriculum change; he was instructed to inform the students.

Some directors mentioned that they had been student representatives themselves and this positively affected the importance they attached to student participation.

These factors and related quotes are summarized in Table 3.

#### Organization of the student representatives

Both stakeholder groups made clear that factors related to the organization of the student representatives have a strong effect on student-staff collaboration. In most schools, student representatives belong to study associations. They meet on a regular basis, usually weekly, in a rather formal fashion to share information, to discuss strategies and to plan activities. Although representatives fulfilled quite different roles in the educational organization (see “Appendix 1”), they often worked together in close relationships. Building support among peers in the student populace, in order to be able to adopt a common opinion and position in their interactions with staff, was considered crucial for the attainment of desired goals. However, it appeared challenging to develop all-inclusive strategies that represented the entire student populace in all its diversity, since part of the student population lacked interest or did not fully understand decision-making outcomes. This made it difficult to act on behalf of the student population and to communicate outcomes. Despite these difficulties, in the questionnaire 75% of the responding representatives expressed the opinion to represent the entire student populace.

Moreover, student representatives and program directors were of the opinion that coordination between student representatives is a prerequisite for collaboration with staff and effectiveness of their actions. A lack of coordination could frustrate these attempts. Some program directors noticed that as a result of a high turnover of student representatives, sometimes there was neither a consistent student policy or shared student opinion, nor any knowledge of major developments and past decisions. However, representatives’ hand-overs usually took place in an elaborate and well-structured fashion. Notably, students carefully recruited and selected their successors in order to find those candidates who fitted the vacant representative’s role best. Similarly, students helped ensure continuity by guaranteeing overlap in appointments of student representatives. Hence, experienced representatives could work alongside new appointees in similar capacities and could coach them during meetings with staff. The other way around, students valued collaborating with staff who had knowledge of their student organization. Program directors stated that their knowledge of students’ organization, roles and responsibilities helped them to set clear expectations of student representation.

Both students and program directors fully agreed that in order to bring benefits, the organization of student representatives should act in the sole interest of students, independent of the educational organization. Aside, most program directors explicitly expressed their support for a selection procedure for student representatives, independent from the school. In particular, students pointed to their role in organizing self-evaluation and coaching sessions among peers as a motivating and supporting factor. Although in most schools they had adopted a practice of evaluation and mutual support, this did not generally occur on a frequent and sustained basis. Students expressed the need for more evaluation and coaching by peers and staff. This was recognized by most program directors.

These factors and corresponding quotes are summarized in Table 4.

**Table 4:** Perceived factors influencing student participation: the organization of student representatives and the school’s organization

Organization of student representatives		
Organization	Strong internal organization Representatives working in close relationships Working with coordinated actions	*“And then you also engage in dialogue with such a policy council like, hey, is it true, we get this back from everyone across all years and can you ask your own members too, like, to give examples. So it also works in the opposite direction, to secure broad endorsement during a policy council, where all committees are represented, for something put forward by a small group. In order to reinforce an argument, for the program to realize like, oh, this is a widely supported [idea].” (FG*-*5)* *“And one student said this and another student said that and yet another student said this, which led to: how can you [speak of] student representation when it is a personal view?” (FG*-*3)*
Support	Building support among peers	*“So we really for sure do have a student base that we represent. We often take polls, that kind of things. […] and we do use our student populace as an argument too, but it really is difficult to reach your student base. There is […] a certain group of students who are very active and a certain group of students who enter through the front door and exit through that same door again.” (FG*-*4)*
Professionalism	Importance of structured hand-overs Independent position Importance of independent representatives’ selection	*“That* [selection of student representatives] *we have left completely to the students. When we need a new student*-*assessor, they [student representatives] organize that themselves. Then the students have a selection procedure. […] No, we are not involved, but we have an introductory meeting with the representative after the selection.” (Int*-*6)*
Need for improvement	Coaching, feedback and evaluation Communication with student populace	*“We just had an internal evaluation and it was really nice to see what the others think of you and everyone really appreciated it. So I have the impression that we were all in a very positive way, re*-*energized by that.” (FG*-*4)*

School’s organization		
Organization	Culture of appreciation and reward Organization with regular, frequent meetings Transparent structure for student representation	*“And that is discussed in an extensive session with teachers and students together. And after that we have a drink together. And all the participation and representation students have a pizza meeting with me as educational director twice a year and then we will just discuss faculty issues in a broader sense.” (Int*-*4)*
Support	(Financial) support and facilitation of student Representatives Appreciation and gratitude	*“[…] and we* [school management] *also have ‘student*-*input*-*thank*-*you evenings’, as they are called, during which each student receives a book gift, a 25*-*euro book voucher, and we enjoy a pizza together and have an informal chat. So we do that a couple of times a year. Yes, and that is how we accommodate it; and it caters to the core, because we take it seriously.” (Int*-*4)*
Professionalism	Importance of clear policy and strategy on student participation Structurally appoint students Offer training opportunities for student representatives	*“Give much more exclusive feedback to students. So a kind of workplace learning, that you make it explicit every once in a while. And that you sit together with the students and say, I have worked together with you for a year now and it has struck me that this and this you do really well. And perhaps there are a couple of opportunities for improvement. I think, that we can do a little better still with one another.” (Int*-*4)*
Need for improvement	Coaching, feedback and evaluation of students Response to student actions No urgent need for changing the level of student participation	*“If you just write a good advice and give some useful advice a couple of times you actually demonstrate that you have something good to say and that you are useful and that you are able to contribute. Then you also notice that managers take you even more seriously, because something really comes out.” (FG*-*4)* *“I do not hesitate to say that we work at all levels, up to participation. I think we have a wish to develop partnership and at the same time we still have cold feet to practice it. I think we are acting at the interface between participation and partnership.”(Int*-*7)*

Quotes are taken from Questionnaires (Q), Focus Groups (FG), and Interviews (Int)

For purposes of enhanced coherence and legibility, quotes, questionnaires and interview scripts have been subject to light editing before journal submission. The authors, however, based their analysis on the original, untidied-up transcriptions, questionnaires and scripts. If necessary, the original quotes can be requested from the first author

### The school’s organization

#### Culture of appreciation and reward

In the questionnaire, a majority (88%) of the responding representatives mentioned to feel positively valued by the school. Gratitude for the contributions of the representatives was often expressed in an informal way at the end of a period, usually by the chair of the committee or council, accompanied with a speech and a small present. All schools organized, facilitated and supported student representation also in a formal fashion, although remarkable differences were observed. First, the extent to which student participation was integrated into the school’s organization differed, although generally there was embedment of representatives at all levels. Second, some schools provided support of student representatives with introduction sessions, training programs, or coaching, whereas other schools neither specifically prepared the representatives for their roles nor supported them by giving feedback on their functioning. Third, in all schools the management provided financial support to students, such as a yearly budget for the organization of student representatives and individual payment. However, the amount and allocation of payment for a particular role differed within and between schools. Most students considered the financial compensation not as their most important motivator, mainly because they perceived the amount as very modest or too low in relation to the efforts required. Fourth, most schools differed in offering a variety of facilities, such as written statements for exemption from certain educational activities, computer equipment, informal meetings with drinks and food, congress visits, and occasionally temporary jobs. The majority (81%) of responding students felt facilitated by the school and students generally agreed that these initiatives made them feel valued. More specifically, provision of physical space for peer discussions and opportunities for training, such as courses in quality assurance and leadership were seen as a token of appreciation for their efforts.

#### Organization, policy and level of student participation

In the eyes of students, scheduled and frequent meetings as well as ad hoc and informal meetings between students and staff are required. In the questionnaire responses, many students (94%) mentioned to provide advice on request and a great majority (80%) of students felt free to give unsolicited advice as well. In addition, student representatives advocated to structurally appoint students to all ad hoc committees and teams for the organization, management and innovation of education.

The involvement of students in organizational decision-making was perceived by both stakeholders as a firm expression of the school’s policy to embrace the students’ views and voices. Students and staff emphasized clarity on the school’s strategy and the importance of putting a supportive culture into practice. Despite the lack of written policies all program directors were very positive on student participation in governance. Most directors supported the engagement of students at all levels of the education program. However, some directors advocated involvement in all domains of governance, while others strongly objected to students having a say in technical projects, financial decisions and examination boards. On the whole, program directors felt more optimistic than students about the degree to which institutional organizations took students’ input seriously, communicated decisions and performed actions. Nevertheless, directors admitted that they did not act on all students’ feedback, essentially because they felt students’ ideas were not always well-informed, or financially or practically feasible.

Finally, our data show that the majority of student representatives were considered to operate at the level of participation. Occasionally, the higher level of partnership was reached mainly by assessors, who operate at the highest level of governance. The actual level of participation depended on conditions such as the individual characteristics of students and staff, and the subject involved. All stakeholders valued the current level of participation as adequate and shared the opinion that student-staff partnership was not in all cases required for effective engagement of students in education governance. These factors and related quotes are summarized in Table 4.

### Value of student participation

Addressing the second research question revealed positive and negative consequences of student participation in governance. Students and directors shared the view that the school highly valued the contributions of student representatives. In the student questionnaire, 74% of responding students sees their participation as important based on their unique and specific experiences and visions. All program directors emphasized the unique views of students regarding the education program and its governance. Student representatives were seen as essential stakeholders in the education program, experiencing the full curriculum and gaining a holistic view of it. Students made observations that were hidden to staff, explained why curriculum elements were successes or failures and contributed to problem solving. Program directors mentioned that these students could keep staff alert, bridge the ‘generation gap’, and function as ambassadors for the school. They appreciated students’ critical and positive attitude and several directors indicated that representatives sometimes acted as allies in advocating changes in the policy and organization of education.

In contrast, negative consequences were observed mainly regarding effectiveness and efficiency. Sometimes, efforts by students did not always result in action by the management. In the questionnaire, not more than half of the responding representatives considered their actions as effective. With respect to efficiency only one-third of responding representatives found their actions efficient. This means that investing considerable amounts of time and energy occasionally have a poor or no result. Students explained that their efforts, depending on the topic of interest, often appeared to be long-term processes or had no visible effect during their appointment as a student representative. Program directors, from their point of view, indicated that participation of students could also cause delays in processes of decision-making and implementation.

Student participation offered representatives opportunities for learning and career. They functioned in a rich learning environment where they participated in professional decision-making in a complex organization and learned how to represent the student voice. Students built and used a network and created relationships with each other and academic staff. Furthermore, personal, professional, and academic development were considered as important benefits for student representatives by all stakeholders, as was also indicated in the questionnaire—of the respondents, 87% reported development of personal and professional competencies, 74% reported development of leadership and 51% reported professionalism and networking. Students in focus groups felt that their ability to collaborate with peers and staff grew with experiences as they learned more about the functioning of complex organizations, leadership, and educational design. They developed useful competences such as communication, strategic and metacognitive thinking, providing arguments, debating, networking, lobbying, and organizing. Directors indicated that student representatives showed competence development and were often seen back in academia.

Learning opportunities were not always experienced optimally. In the questionnaire, 45% of the respondents mentioned to feel individually supported, whereas 48% reported not to feel supported. In the focus groups, representatives indicated to value a personal touch from staff members by direct feedback on students’ activities, as well as informal personal and professional counselling, which gave them recognition and fueled their motivation and collaborative aspirations. However, a lack of feedback, coaching or response to actions resulted in demotivation of representatives.

Perceived values of student participation and corresponding quotes are summarized in Table 5.

**Table 5:** Perceived values of student participation and student-staff collaboration: positive and negative consequences for the school and student representatives

Positive consequences for the school		
Students as unique stakeholders in education program	Experience and holistic view of curriculum Reliable, detailed evaluation of curriculum	*“Only as a student you progress through six years of the curriculum and so you see everything. Well, there is no teacher, apart from his own background, who is that familiar with the curriculum. So in that sense, you would be a fool if you don’t […] use that to your advantage actually.” (Int*-*1)*
Student representatives as unique stakeholders in governance of education	Observations that are hidden to staff Explanation why curriculum elements are successes or failures Contribution to solving problems Bridging the generation gap Keeping staff alert Contribution to attain goals shared with school Ambassadors for school Valuable for school after representation or graduation	*“But then, when they are graduated, they know everything of my curriculum and sometimes they want to learn something more about curriculum development and all that, so by now I have five active, five medical school graduates* [currently employed]*. But that is actually the team on which my curriculum committee very much pivots.” (Int*-*2)* *“They* [students] *think in other frameworks. They can much easier think out of the box and come up with smart, creative solutions. Of course* [those solutions are] *based on their interest, but also based on their youth, another phase of life, and having another input and output.” (Int*-*10)*

Positive consequences for student representatives		
Opportunities for learning	To function in a rich learning environment To participate in professional decision-making To learn to represent student voice and view To build and use a network To develop professional relationships with academic staff	*“But I miss some coaching [..], just you and […] a professor or some other educationalist or someone just not that much involved directly, sitting down, relaxed, who just looks at where you are now and what do you want, to bring you back for a minute.” (FG*-*5)*
Developing personal, professional and academic competences	Communication, collaboration, organization Advocating, lobbying, arguing A critical attitude, metacognitive thinking Insight in functioning and governance of a complex, professional organization	*“[…] assertiveness and lobbying, those are also actually pretty beautiful aspects of personal development that you hope to master in such a year..” (FG*-*5)*

Negative consequences for the school		
Inefficiency and delay	Delays in processes of decision-making and implementation	*“One of the problems is that the various governance platforms usually have not discussed their points of view with each other. With the Education Committee you go in one direction, whereas the Faculty Council wants something different. […] Now we are trying to better tune the agenda and provide the information just*-*in*-*time to all participants in governance in a joint effort of students and staff.” (Int*-*7)*

Negative consequences for student representatives		
Ineffectiveness and inefficiency	Efforts do not always result in effect or improvement High investment, low outcome	*“For example, we had many complaints about the new governance model; we have written many letters and spoke to many different people. Finally something happens and it has a result, but it takes a very long time. On the other hand, changing is hard; I don’t think it can be done with more efficiency.” (FG*-*1)* *“You know what you want to do. You know what you want to try to solve it. Then you are sent in all directions without getting an answer, it gets later and later. These are the things that eventually are going to get itchy.” (FG*-*3)*

Quotes are taken from Questionnaires (Q), Focus Groups (FG), and Interviews (INT)

For purposes of enhanced coherence and legibility, quotes, questionnaires and interview scripts have been subject to light editing before journal submission. The authors, however, based their analysis on the original, untidied-up transcriptions, questionnaires and scripts. If necessary, the original quotes can be requested from the first author

## Discussion

Our findings show that student participation in governance is influenced by characteristics of the individual student and staff member, the organization of the student representatives and the school’s organization. This myriad of contextual influences can either stimulate or hinder effective student participation. This broad set of factors and its complexity is consistent with the conceptual model of Kahu (2013) and the findings of Lizzio and Wilson (2009) and Carey (2013). The recent model proposed by Carey (2018) emphasizes the key role for institutional action in facilitating student engagement in university decision-making. Our results do not only emphasize the role of the institute, but also show the crucial impact of the student representatives’ organization on student participation in education governance.

Most, if not all of the individual student characteristics can be considered as personal, professional and academic competencies that are vital for an adequate performance of a representative’s role. These competences are also relevant for students’ professional careers and social functioning (Carey 2013; Dickerson et al 2016; Lizzio and Wilson 2009) and are in line with competency frameworks, which are generally applied to educate medical and veterinary students (Bok et al. 2011; Frank and Danoff 2007). Schools as well as organizations of representatives should be aware of the importance to create learning opportunities for students and guide them to develop these competencies.

The attitude of individual staff has a major influence on the collaboration with students, as was pointed out in several reports (Carey 2013; Lizzio and Wilson 2009; Planas et al. 2013). Several inhibiting characteristics, such as the resistance or ignorance by individual staff (Bovill et al. 2016; Carey 2013; Lizzio and Wilson 2009), were observed in our study. Remarkably, power distance as reported by e.g. Stalmeijer et al. (2016) did not seem to be a barrier for student participation in governance. This is presumably related to the intensive and prolonged collaborations of staff with highly-motivated and well-organized student representatives, as demonstrated in our results.

A number of strategic and coordinating actions by student organizations were found crucial for successful and effective student participation. First, the independent position of the student representative organization, which is advocated by directors and students alike. Second, the student organization carefully selects candidates using their own independent procedures, based on motivation and competences. This is in contrast to student representatives with democratic and political ambitions, who are elected by the student populace to act as influential student movements in shaping higher education policy (Klemenčič 2012, 2014; Luescher-Mamashela 2013). Based on our results, we conclude that independent selection of students by the student organization is a successful strategy to attain optimal student participation in governance of education. Hence, there is no need for the schools to wrestle with a problem they can hardly solve: matching student profiles with requirements for representative roles. Selection procedures may also avoid role ambiguity, reported by Lizzio and Wilson (2009) to be the greatest challenge for student representatives. We found that thanks to well-organized student representatives, despite the lack of a formal policy, the positions, tasks and responsibilities of student representatives in the school’s governance structure are known by involved student representatives. However, there was a call for a formal framework for student participation, as advocated by Peters et al. (2018). Third, a well-planned hand-over and coaching by experienced representatives is crucial when new candidates take up their role, since turnover of student representatives in most roles is rather high and was perceived as a barrier by the stakeholders. Fourth, regular coordination of strategies and actions using a platform of representatives is a prerequisite for effective student participation in governance. Essential elements in coordination are recognized: obtaining and sharing information, discussion and development of opinions and strategies, concerted planning of activities, collaborating with peers or staff, followed by debriefing and evaluation of the activities. Fifth, contacts and communication (‘closing the loop’) with the student populace are important to make sure that the student voice is adequately represented and that all students are informed about the outcome of student participation. However, this was considered a challenge, in line with experiences of student evaluation in quality assurance (Griffin and Cook 2009).

Although distinct levels of student engagement were recognized, it is remarkable that the majority of student representatives were considered to operate at the level of participation and the higher level of partnership was reached only occasionally (Healey et al. 2014). According to the stakeholders student-staff partnership was not absolutely necessary, in line with the report of Bovill and Bulley (2011).

The value of student participation in governance is described in terms of benefits for the university, the student and the society (Lizzio and Wilson 2009). In our study, the stakeholders identified as major benefits for the school the quality and governance of the education program, and as benefits for the representatives the personal development, learning opportunities and recognition. It should be emphasized that governance covers a broad domain, and student participation is valued particularly in those areas, where students are unique stakeholders such as in the education program, student facilities, schools’ policy and strategy. Several benefits for student representatives found in this study, like opportunities for learning and development of competencies, were reported earlier by Lizzio and Wilson (2009) and Carey (2013). These generic benefits seem to apply to different settings of student representation, irrespective of the type of study.

Negative consequences of student participation appear to be low effectiveness and efficiency of students’ efforts. These may result in frustration and loss of motivation, and can ultimately jeopardize potential benefits for students as well as the school. While students indicated that their efforts were often long-term processes, program directors mentioned that participation of students could cause delays in decision-making and implementation. These negative consequences were not reported earlier (Visser et al. 1998, Lizzio and Wilson 2009).

In all veterinary and medical schools a culture of support, facilitation and reward is experienced by students and stimulated by directors and coordinators in educational programs. However, there is a clear need for improvement of individual student coaching and feedback.

## Strengths and limitations

A strength of this study is the empirical, qualitative multi-method approach. An explorative questionnaire was used to prepare for in-depth discussions in focus groups and also to obtain quantitative support for relevance of issues. A second strength is the identification of many themes shared by the stakeholders, over a wide range of activities in student-staff participation, across a miscellany of institutions. This variety allowed to obtain a more comprehensive understanding of student participation in terms of the associated organizational culture and structure, the benefits and the constraints. The results may be transferable to an international context or to student engagement in other settings, taking into account possible structural and cultural differences. In this regard we emphasize that in the Netherlands, student participation is mandated by law. However, this applies only to faculty and university councils and for education committees aiming at quality assurance of the education program, but not for management teams and ad hoc committees.

A potential limitation relates to the choice of program directors as stakeholders instead of other staff. For the purpose of our study, however, program directors were deemed most appropriate to put staff experiences into a broader, managerial perspective. Three authors (SM, JvV, AdG) have been directly involved in student participation in governance and potential preconceptions may have interfered with the facilitation and interpretation of data. On the other hand, their experience and familiarity with the context, terms and concepts discussed could allow them to identify meaningful experiences.

## Main conclusions and implications for practice and future research

This study underscores the value of student participation in governance for the education program, the school and the student representatives themselves. Although it requires investments in time and energy, students are found to be valuable stakeholders in governance and they develop personal, professional and academic competences, in line with aims of competency-based education. Based on the positive experiences with regard to the quality and continuity of student representatives we advise to let students select their own successors based on motivation and competences.

We want to point out the expressed (students) and recognized (directors) need for a proper introduction and well-planned student handovers, training, coaching and personal feedback for student representatives. Therefore, it is vital that student participation is nested in a supportive professional network, both within their own student organization and within the schools’ organization. We have witnessed the importance of a cohesive student organization, where student representatives regularly meet (from once a month up to once a week). When coordinated well, students can inform, discuss opinions, strategies and tactics and learn with each other, ultimately leading to a concerted planning of activities.

Unique benefits can be effectively derived from student participation, but this requires schools to provide a transparent organizational structure creating opportunities for student-staff collaboration. Furthermore, it is important to foster a culture that welcomes and coaches students to engage in the school’s governance. We suggest that schools adopt a clear, shared policy on student participation, do not impose students’ participation on staff, but rather inform and inspire them to empower student participation in governance.

Observational and comparative research designs are of interest to further support or challenge students’ contribution to governance processes. Educational researchers may additionally work on the development and effects of interventions focusing on student participation, student-staff collaboration and coaching. More specifically, methods to increase learning opportunities ‘on the job’ and empower talented students to develop personal, professional and academic competences should be further explored. Lastly, we encourage international research into school culture and organization, and individual staff attributes with respect to student participation.

## Appendix 1: Positioning and roles of Dutch student representatives

**Table:**

Student engagement is legally enshrined in the Higher Education and Research Act which specifies that students should be represented at three levels: the university council, the faculty council or student council and the education committee of their curriculum (Act on Higher Education and Scientific Research (in Dutch: Wet op het hoger onderwijs en wetenschappelijk onderzoek, WHW) 1992). The faculty council or student council acts in the school’s governance and policy. The education committee has an advisory function in assuring the quality of education in bachelor’s and master’s programs and, according to Dutch law, consists of fifty percent staff and fifty percent students.
Apart from serving on these bodies, student representatives may fulfil many other roles, depending on the respective medical or veterinary school. For instance, some act as advisors to the executive board of the university medical centre or to the medical or veterinary education program directors and their management teams. Other students participate in ad hoc committees for curriculum design or renewal, for student facilities, for education policies, etc. In the Netherlands, each medical and veterinary school has appointed a student assessor who represents the students at the highest level and who is in direct contact with the program director and sometimes the dean or even the executive board of the university medical centre. Most student assessors fulfil a (nearly) full-time job and have a good overview of the activities undertaken by their peer student representatives.

## Appendix 2: Qualitative questionnaire

### Title of the study

Student engagement in medical and veterinary schools in the Netherlands

### Introduction

Dear student,

With this letter, we would like to invite you to participate in a study on the engagement of students in medical and veterinary schools in the Netherlands.

The study will be conducted by Stephanie Meeuwissen (sixth-year medical student at Maastricht University), Jeroen van Veen (fifth-year medical student at Maastricht University), Dr Annemarie Spruijt (Department of Educational Development and Research, Maastricht University) and Prof. Ton de Goeij (Institute for Education, FHML, Maastricht University).

We are contacting you for this study because you have experience as a student representative.

The study encompasses the completion of a survey and/or participation in a focus group and will take place at a location of choice. With this information letter, we invite you to participate in the survey. We will contact you for participation in the focus group at a later date.

Whether you participate in the study or not will not affect the evaluation of your educational role or your personal educational achievement in any way. The study results cannot be traced back to individuals or faculties, nor will they affect your personal educational achievement or educational role.

We would like to ask you to read the information below carefully. Should you agree, we ask you to complete the informed consent form on the first page of the electronic survey.

1. **What is the study purpose?**
  Much has changed in students’ position and role in recent decades. A shift has taken place from the student as a passive consumer of education offered to the student who learns actively and engages with his or her study program. With this study, we would like to learn more about the organization, effectiveness and impact of student representation in medical and veterinary schools in the Netherlands.
2. **What will we study?**
  We would like to study the performance of student representation. In doing so, we would like to identify opportunities for improvement and best practices and probe into the effect of student representation on the personal development of the students involved. We are interested in relevant factors in the organization of student representation, changes made to education and interactions with staff and students in order to shed light on the workings of student representation and on how it achieves results.
3. **How will we execute the study?**
  We will execute the study by using surveys and focus groups at all medical and veterinary schools in the Netherlands. This will take place in the period from April to September 2016. The study will also focus on staff members who are responsible for the organizational structure of the study program. We will interview these stakeholders about the organizational structure, input and efficiency of student representation and about how this bears on the quality of the study program.
4. **What do we expect from you?**
  We want to ask you if you are willing to participate in the study by completing the survey, which will take about 20–30 min of your time.
5. **What risks could be involved?**
  There is no physical or psychological risk during the study. The decision to participate in the study or not will not affect the evaluation of your personal educational achievement or your role in education in any way. The study results will not affect the evaluation of your personal educational achievement or your educational role either.
6. **What advantages and disadvantages could my participation in this study potentially have?**
  A disadvantage of the study is the time it takes to participate in the study.
7. **What happens if I do not wish to participate in this study?**
  Participation in this study is voluntary. It is up to you to decide whether to participate in the study or not. If you decide not to take part, you do not need to do anything. You do not have to sign anything either, nor do you have to indicate why you do not want to participate. If you do participate, you can always change your mind and opt out at any time. Should you decide to cancel your participation, we will destroy the data related to your participation.
8. **Will you inform me if new information about the study will become available during the course of the study that is relevant to me?**
  Should any relevant information become available in the course of the study, you will be informed.
9. **What happens to my data?**
  All the information we collect by means of surveys and focus groups is confidential. Only the aforementioned researchers shall have access to this information. Before we publish the information, we will delete all references to your personal identity or to the faculty where you study. It is possible that we use one or more of your comments to illustrate a general theme. However, we will never link your proper name to any of these comments.
10. **Will I receive compensation for participation in this study?**
  We offer all participants a workshop on student engagement during the annual national medical student conference (LMSO). Participants in this study will be given priority when they register for this workshop.
11. **Did the ethical review board of medical education research approve this study?**
  We obtained ethics approval for this study from the Ethical Review Board of the Netherlands Association for Medical Education (NVMO).
12. **Questions**
  You can address further questions about the study at any time to:
  Stephanie Meeuwissen: [telephone number] or s.meeuwissen@student.maastrichtuniversity.nl

### General

**Table:**

At which faculty do you study?
Email address
Which year of the study program are you in?
How long have you been involved in student participation?
What is your current role in student participation?
How many hours per week do you spend on activities related to student participation?

### Organizational structure

**Table:**

Please indicate for each of the participation targets (a, b, c) how students are involved. Specifically name committees (e.g. Faculty Council, block planning group) and student roles (e.g. assessor, education commissioner). A. Evaluation and quality improvement; B. Change or reform of curriculum content; C. Educational board, management and organization
Evaluation and quality improvement
Reform and change of curriculum content
Educational board, management and organization
Student representative bodies are organized in different ways, both locally and nationally
In what ways do student representatives act in coordination?
In what way are you being prepared for your role as a student representative?
All faculties have a medical faculty/study association. The relationship with student participation appears to vary widely across faculties
Is an organization such as the faculty/study association involved in student participation?
How are students recruited and appointed to different positions?
As a token of appreciation, the university usually supports student roles. In what ways does the university reward students who are involved in student participation?
To what extent do you feel valued as a student representative by the educational organization? How does this show?
In what way does the educational organization/faculty facilitate and/or support student representation?
What do you think is your faculty’s vision when it comes to student participation?

### Effectiveness of student participation

**Table:**

How does the educational organization capitalize on your participation?
To what degree do you represent your student base and please explain this
In what way do you report what you accomplished as a student representative back to your student base?
How effective is student participation at your study program and how do you explain this?
How efficiently has student participation been organized?
Could you indicate the importance of student participation to each of the targets mentioned below and why? A. Evaluation and quality improvement; B. Change or reform of curriculum content; C. Educational board, management and organization
What did you achieve by means of student participation? How did you manage to achieve this? Please give an example
What points were you not able to realize in terms of actual results and how do you explain this?

### Improvement of student participation

**Table:**

Are there any factors that constrain or inhibit student participation at your study program? How do you explain this?
How could student participation be improved at your study program?

### Best practices

**Table:**

What factors have led to your success? (From the perspective of both the student and educational organization)
List one or two ‘best practices’ concerning student participation at your faculty and please explain this

### Personal and professional development

**Table:**

What was your personal motivation to become a student representative?
What is the effect of your role as a student representative on your personal and professional development?
Do you receive support to facilitate your learning experience? (In the form of evaluation meetings or trainings, for example)

### Closure

**Table:**

Do you have anything to add or any comments in response to this questionnaire?	
